# Hierarchical Porous LiNi_1/3_Co_1/3_Mn_1/3_O_2_ Nano-/Micro Spherical Cathode Material: Minimized Cation Mixing and Improved Li^+^ Mobility for Enhanced Electrochemical Performance

**DOI:** 10.1038/srep25771

**Published:** 2016-05-17

**Authors:** Zhen Chen, Jin Wang, Dongliang Chao, Tom Baikie, Linyi Bai, Shi Chen, Yanli Zhao, Tze Chien Sum, Jianyi Lin, Zexiang Shen

**Affiliations:** 1 Energy Research Institute (ERI@N), Interdisciplinary Graduate School, Nanyang Technological University, Research Techno Plaza, 50 Nanyang Drive, 637553, Singapore; 2 Division of Physics and Applied Physics, School of Physical and Mathematical Sciences, Nanyang Technological University, 21 Nanyang Link, 637371, Singapore; 3 Energy Research Institute (ERI@N), Nanyang Technological University, 50 Nanyang Drive, 637553, Singapore; 4 Division of Chemistry and Biological Chemistry, School of Physical and Mathematical Sciences, Nanyang Technological University, 21 Nanyang Link, 637371, Singapore; 5 School of Materials Science and Engineering, Nanyang Technological University, 639798, Singapore

## Abstract

Although being considered as one of the most promising cathode materials for Lithium-ion batteries (LIBs), LiNi_1/3_Co_1/3_Mn_1/3_O_2_ (NCM) is currently limited by its poor rate performance and cycle stability resulting from the thermodynamically favorable Li^+^/Ni^2+^ cation mixing which depresses the Li^+^ mobility. In this study, we developed a two-step method using fluffy MnO_2_ as template to prepare hierarchical porous nano-/microsphere NCM (PNM-NCM). Specifically, PNM-NCM microspheres achieves a high reversible specific capacity of 207.7 mAh g^−1^ at 0.1 C with excellent rate capability (163.6 and 148.9 mAh g^−1^ at 1 C and 2 C), and the reversible capacity retention can be well-maintained as high as 90.3% after 50 cycles. This excellent electrochemical performance is attributed to unique hierarchical porous nano-/microsphere structure which can increase the contact area with electrolyte, shorten Li^+^ diffusion path and thus improve the Li^+^ mobility. Moreover, as revealed by XRD Rietveld refinement analysis, a negligible cation mixing (1.9%) and high crystallinity with a well-formed layered structure also contribute to the enhanced C-rates performance and cycle stability. On the basis of our study, an effective strategy can be established to reveal the fundamental relationship between the structure/chemistry of these materials and their properties.

With the increasing global energy demand, the rechargeable lithium-ion batteries (LIBs) have been intensively investigated as powerful sources for electric vehicles (EVs) and hybrid electric vehicles (HEVs) over the past few years^1^. LiCoO_2_ (LCO), which was firstly reported by the Goodenough group in 1980^2^, was commercialized by Sony Corporation in 1991^3^. However, LCO suffers from many detrimental shortcomings, such as low thermal stability, limited specific capacity, high cost and environmental toxicity^2^,^4^. In 2001, the pioneering work by Ohzuku and Makimura^5^,^6^,^7^ enabled the successful implementation of LiNi_1/3_Co_1/3_Mn_1/3_O_2_ (NCM) which has the same layered α-NaFeO_2_ structure with overall hexagonal symmetry (space group *R-3m*) as LCO^8^ but with much higher capacity, better thermal stability, more environmental benign and lower cost^4^,^9^,^10^,^11^. However, NCM still suffers from two notable shortcomings, *i.e.* the fatal capacity degradation in long cycles and poor rate capability especially at high C-rates. These serious problems can be attributed to the following reasons: 1) the relatively low conductivity; 2) the anti-site mixing between Li^+^ (0.76 Å) in the Li^+^ layer and Ni^2+^ (0.69 Å) in the nearest Ni-O layer, which is thought to be a thermodynamically favored feature that will depress Li diffusion and lead to the deterioration of electrochemical performance, especially the C-rates capacity; 3) the irreversible side reactions which occur with electrolyte at high voltages, usually accompanied with the loss of oxygen.

The synthesis route and conditions of NCM (*e.g.* starting materials, chemical composition and crystallization procedures) have significant influence on the phase purity, crystal size, particle morphology, surface area and cation mixing, which further affects the electrochemical performance^6^,^12^. Traditionally, NCM can be prepared by various methods^13^,^14^,^15^,^16^,^17^,^18^,^19^, including co-precipitation, solid-state reaction, hydrothermal, spray-drying, sol-gel, molten salt method and microwave treatment etc. The co-precipitation method is one of the most commonly used methods to obtain NCM, due to its advantages such as relatively uniform granularity, easily controlled process, lower synthesis temperature and ease for industrial scale-up. However, it is still a challenge to control the process complexity and improve reaction stability of the wet chemical synthesis. Solid state reaction is another common synthesis route for NCM. The method is simple and the process is easily controlled. However, it requires mechanical mixing and refinement of the raw materials. Therefore, it is difficult to obtain the product with homogeneous cation distributions, which significantly influences the electrochemical performance. Recently, a template sacrificial method has been used to obtain materials with controlled morphology. In a typical approach, the Li, Ni, Co precursor materials are mixed well with porous micro-sized fluffy MnO_2_ as the template. Subsequent high temperature calcination leads to the formation of NCM with hierarchical porous nano-/microsphere structure^20^,^21^,^22^. Li diffusion kinetics in nano-sized particles and particle surfaces are found to be less sensitive to the presence of anti-site defects^23^. In addition, the nano-sized primary particles in nano-/micro hierarchical architecture can not only shorten the lithium ion diffusion path, but also minimize the unwanted anti-site cation mixing, greatly improving the Li diffusion kinetics. On the other hand, the presence of micro-sized secondary particles which inherit their size/shape/basic-morphology from porous fluffy MnO_2_ template can increase the initial Coulombic efficiency and stabilize the structure during charge/discharge processes. Furthermore, such two-step process is very suitable for commercial fabrication^24^.

Herein, we present our work on a highly porous hierarchical nano-/microsphere LiNi_1/3_Co_1/3_Mn_1/3_O_2_ (PNM-NCM) with extremely uniform size distribution and controlled morphology that is synthesized by a sacrificial template method. The HRTEM and XRD results show the high crystallinity of the prepared PNM-NCM with excellent hexagonal ordering and low anti-site cation mixing, while SEM and BET measurements reveal a highly porous framework with a large surface area. Cyclic voltammetry (CV) studies reveal minimum polarization and ~100% reversibility of charge/discharge, confirming the extremely low cation mixing in our sample. In addition, the Li mobility is also found to be obviously improved as revealed by the CV studies at various scan rates. Benefiting from this hierarchical porous nano-/micro structure, minimized cation mixing and improved Li mobility, our PNM-NCM sample exhibits significantly improved electrochemical performance as compared to conventional Bulk-NCM for advanced LIBs. The cell made of PNM-NCM microspheres delivers high specific reversible capacity of 207.7 (0.1 C), 178.4 (0.5 C), 163.6 (1 C), 148.9 (2 C) and 120.0 mAh g^−1^ (5 C), respectively. The reversible capacity retention is as high as 90.3% after 50 cycles. In this paper, the correlation between fabrication conditions and structure properties of PNM-NCM microspheres is also discussed.

## Results and Discussion

**Figure 1:**
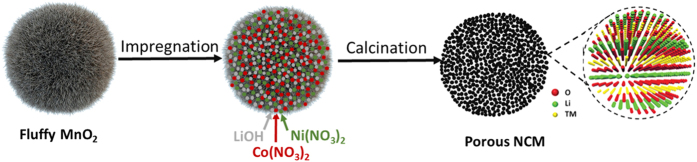
Illustration of synthesis route of PNM-NCM microspheres.

In order to obtain highly porous hierarchical nano-/microsphere LiNi_1/3_Co_1/3_Mn_1/3_O_2_ (PNM-NCM), we designed a simple and facile two-step method using porous fluffy MnO_2_ as the sacrificial template as well as the Mn source in the first step. In the second step, nickel nitrate, cobalt nitrate and lithium hydroxide were introduced by impregnation, followed by high temperature calcination at 900 °C for 12 h. This synthesis route is schematically demonstrated in Fig. 1.

**Figure 2:**
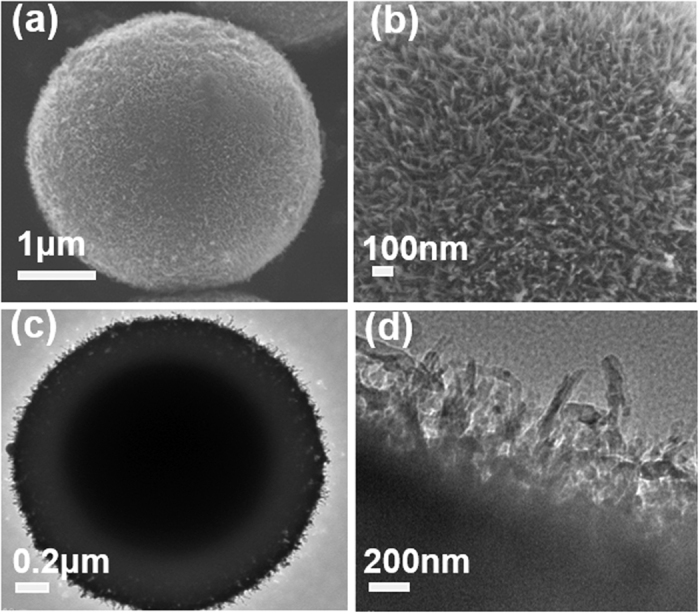
Typical (**a,b**) FESEM images of porous fluffy MnO_2_ at different magnifications; (**c,d**) TEM images of porous fluffy MnO_2_ surrounded by short nanowires at different magnifications.

The as-prepared porous fluffy MnO_2_ has uniform fluffy spherical morphology as shown in Fig. 2a,b. The particle size of porous fluffy MnO_2_ is well distributed, around 3–5 microns in diameter (Fig. S1†). From TEM images (Fig. 2c,d), MnO_2_ is surrounded by short nanowires in the surface. Besides, this fluffy MnO_2_ is very porous, and BET measurements show its pore size is around 1.9 nm with a surface area of 245.8 m^2^ g^−1^ (Figs S2 a† and S2 b†). The porous structure and micro-size of as-prepared fluffy MnO_2_ are very favorable for serving as a sacrificial template for the subsequent solid state reaction with Li-, Ni- and Co- chemicals. The narrow-distributed pore size (1.8–2.1 nm) is ideal for the impregnation of Ni, Co and Li ions, their homogeneous mixing and the subsequent stoichiometric solid state reaction.

**Figure 3:**
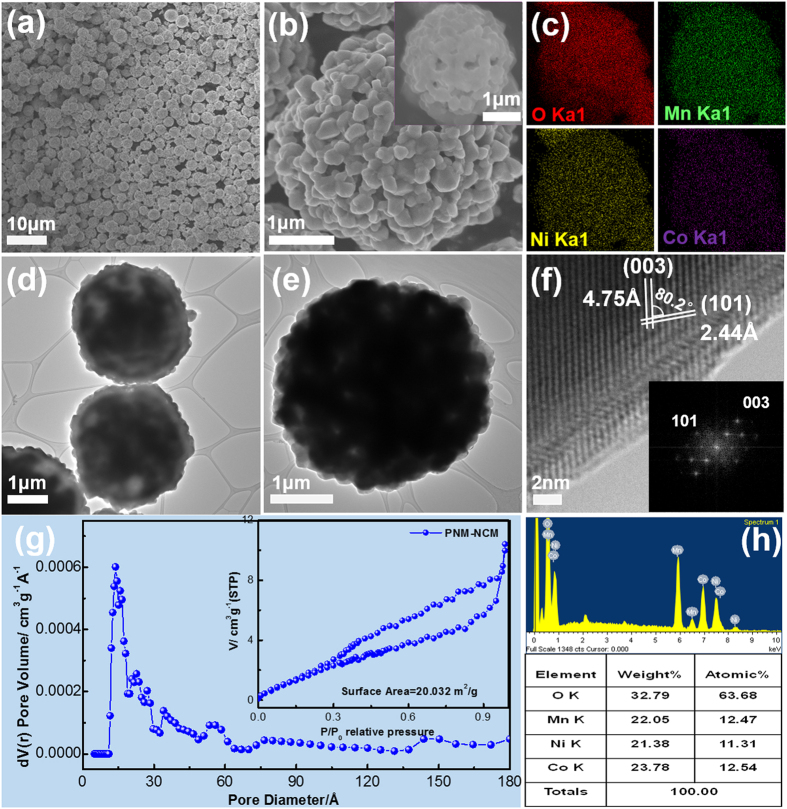
Typical FESEM images of (**a,b**) PNM-NCM at different magnifications; (**c**) EDS mappings of oxygen, manganese, nickel and cobalt; (**d–f**) TEM and HR-TEM images of PNM-NCM; (**g**) the pore size distribution, and N_2_ adsorption/desorption isotherms of PNM-NCM; (**h**) EDS spectrum and results of PNM-NCM microspheres.

The PNM-NCM microspheres obtained after calcination has an intact spherical morphology with uniform particle size (around 3 μm as shown in Fig. 3a,b). The as-prepared samples have no sign of agglomeration, which is considered very helpful for obtaining improved electrochemical performance. The TEM image confirms the porous structure as shown in Fig. 3d,e. The lattice spacing of 0.47 nm and 0.24 nm can be indexed to the (003) and (101) lattice planes respectively (Fig. 3f). The FFT electron diffraction pattern in Fig. 3f inset clearly shows the single crystalline nature of the primary NCM particles, indicating the high ordering of the atomic arrangement. Li ions in NCM are known to move preferably along the two-dimensional a-b plane^25^. The (003) plane is perpendicular to c-axial direction and parallel to hexagonal a-b plane. The high ordering of hexagonal crystalline plane in our PNM-NCM is one of the primary criteria for excellent Li ion mobility. The porous structure is also confirmed by BET results (Fig. 3g), where the specific surface area is 20.0 m^2^ g^−1^ and the pore size is about 1.6 nm. The specific surface area of PNM-NCM is much higher than that of Bulk-NCM (only 1.7 m^2^ g^−1^ as shown in Figs S3 a† and b†). This type of hierarchical porous nano-/micro spherical structure is known to favor of Li ion insertion/extraction. It can shorten the Li^+^ diffusion path, alleviate volume expansion/compression during charge/discharge processes and facilitate excellent electrolyte penetration. In order to investigate effect of molar ratio of Ni: Co: Mn as well as the elemental distribution of O, Ni, Co and Mn over the spheres, the EDS mappings (Fig. 3c) and EDS region scan (Fig. 3h) were both performed. The elemental distributions of O, Co, Ni and Mn are homogenously distributed as can be seen from Fig. 3c. The molar ratio of Ni: Co: Mn is around 1: 1: 1 as shown in Fig. 3h.

**Figure 4:**
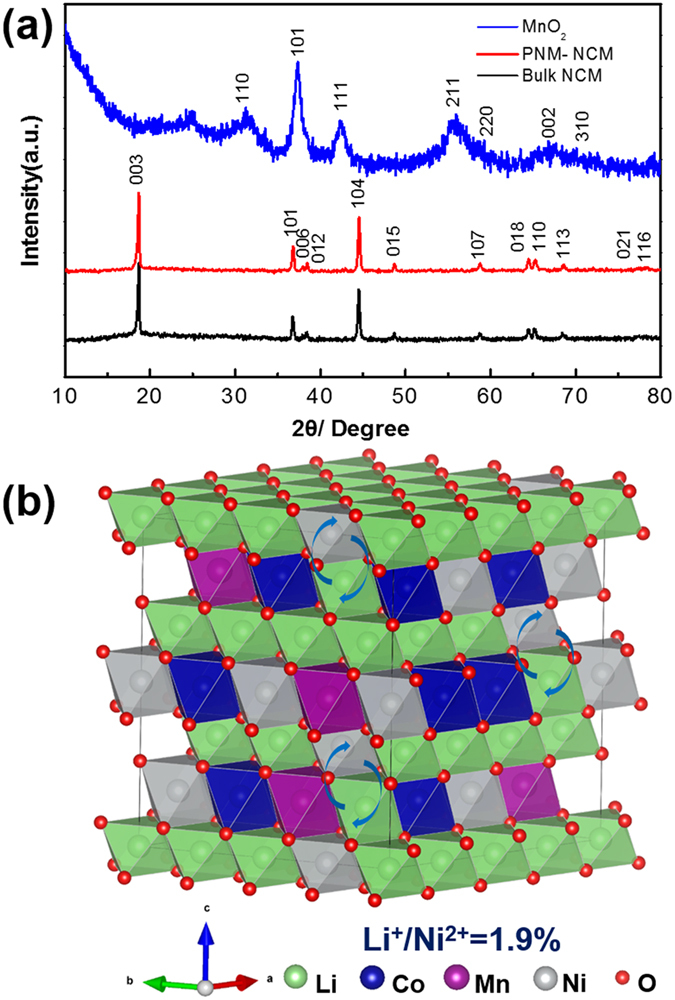
(**a**) XRD patterns of MnO_2_ (blue curve), PNM-NCM (red curve), (black curve); (**b**) the illustrative crystallographic structure of PNM-NCM.

Figure 4a displays the XRD patterns of porous fluffy MnO_2_ PNM-NCM and Bulk-NCM, respectively. All the XRD reflections in MnO_2_ are well fitted to a pure tetragonal single-phase MnO_2_ (JCPDS No. 24-0735), while the pure hexagonal α-NaFeO_2_ (space group, *R-3m, a* = *2.860 Å, c* = *14.216 Å* shown in Supplementary Table 1†) structure is identified in each NCM XRD curve, corresponding to the ideal LiMO_2_ layered structure. The obvious splitting of the (006)/(102) peaks and (108)/(110) peaks is observed both in PNM-NCM and Bulk-NCM, revealing both of them have good crystalline ordering in the hexagonal layers. The R-factor (R = (I_006_ + I_102_)/I_101_) is calculated to be 0.52 for PNM-NCM and 0.58 for Bulk-NCM. (see Supplementary Tables 1† and 2†). R-factor is inversely proportional to the hexagonal ordering, and hence the lower R-factor of PNM-NCM indicates higher hexagonal ordering compared to that of Bulk-NCM. Additionally, the intensity ratios of I(003)/I(104) of both samples are larger than 1.2 (1.399 and 1.417 respectively), indicating the cation mixing between Li^+^ and Ni^2+^ is low. The cation mixing in PNM-NCM microspheres calculated by XRD Rietveld refinement analysis is only 1.9% (see Supplementary Table 1†), indicating the cation mixing is almost negligible. In fact, according to the previous results published by X. Zhang *et al*.^26^, a sample with anti-site mixing less than 2% does not show evident influence on the electrochemical performance. However, Bulk-NCM shows higher cation mixing from XRD results (4.3% as shown in Supplementary Table 2†). Figure 4b shows the illustrative crystallographic structure of PNM-NCM.

Beside of the above mentioned excellent crystalline ordering, small particle size also can attribute to the minimized cation mixing of Li^+^/Ni^2+^. As reported^23^, that Li diffusion kinetics in small particles and particle surfaces is less sensitive to the presence of anti-site defects. The grain size of PNM-NCM (56.6 nm) as determined by XRD is much smaller than that of Bulk-NCM (97.0 nm), leading to lower Li^+^/Ni^2+^ anti-site disorder. When the porous microspherical MnO_2_ is employed as the sacrificial template, it results in a nano-/micro hierarchical architecture for our final product, in which suitable nano-sized primary particle is retained, enabling excellent Li mobility as well as the minimized cation mixing of Li^+^/Ni^2+^ while the micro-sized spherical-shaped porous 3D structure can effectively prevent the primary particles from agglomeration besides other advantages.

All the above mentioned structural properties (parameters), such as grain size and crystalline ordering (peak splitting of (006)/(102) and (108)/(110), cation mixing fraction and R-factor) which will directly affect the electrochemical performance, are closely correlated to the synthesis procedures and conditions. Among others, the calcination temperature is found to be significantly important in controlling the particle size and the crystallinity. We have carefully studied the samples calcined at various temperatures, 800, 850, 900 and 950 °C. As shown in Fig. S5†, sintering at 900 °C for 12 h is the optimum calcination condition, resulting in the most uniform and well distributed grain size, porous microsphere morphology, low cation mixing and high crystallinity. At lower calcination temperature (750–850 °C) the particle size is smaller, which is in favor of lowering cation mixing but is unfavorable for crystalline ordering (in hexagonal a-b plane). If the sample is sintered at very high temperature (>950 °C) the particle size approaches hundreds nm (see Fig. S5h†) and the increased cation mixing will result in low Li ion mobility and thus poor battery performance. This trend is well consistent with previous reports in which 850–900 °C calcination with 50–60 nm particle size is crucial in enhancing rate and cycling performance^24^.

**Figure 5:**
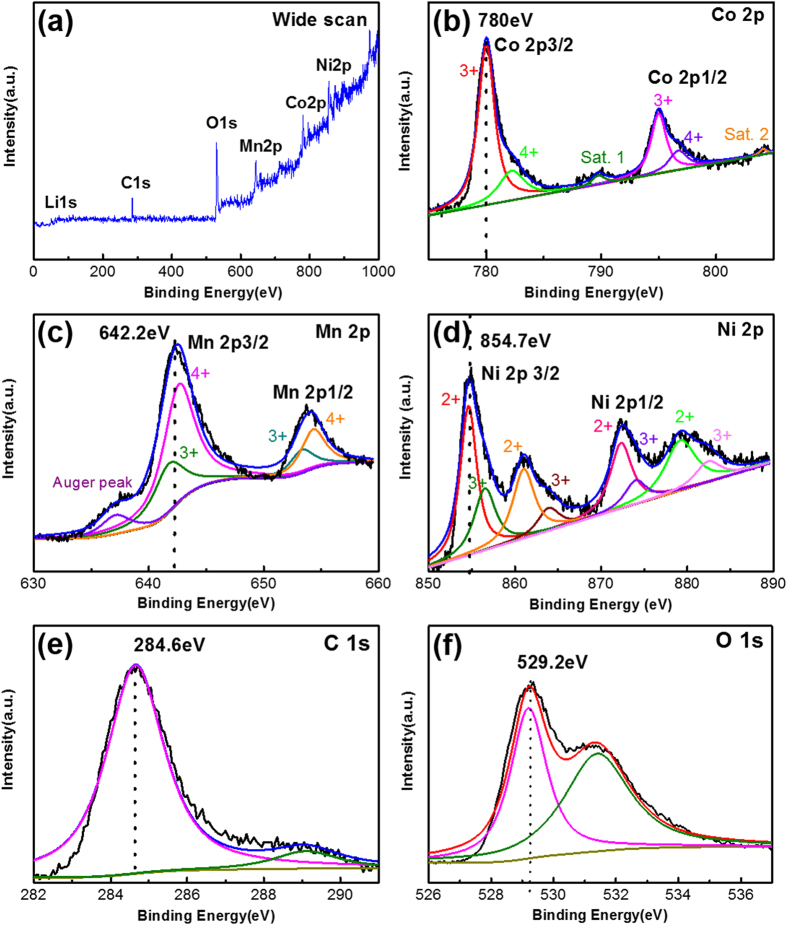
XPS spectra of (**a**) a wide scan, (**b**) C 1s, (c) O 1s, (**d**) Co 2p, (**e**) Mn 2p and (**f**) Ni 2p of PNM-NCM microspheres.

In order to investigate the oxidation states of nickel, cobalt and manganese, X-ray photoelectron spectroscopy (XPS) measurement was performed. The binding energy of C 1s (284.6 eV, as shown in Fig. 5e) was used as the calibration of all the other spectra in XPS analysis. The wide scan spectrum (Fig. 5a) indicates only the presence of Li, C, O, Ni, Co and Mn without any other impurities. From Fig. 5b–d, the binding energy of Co, Mn and Ni at 780.0, 542.2 and 854.7 eV, are corresponding to Co^3+^, Mn^4+^ and Ni^2+^ respectively. The Co 2p spectrum has two main peaks (Fig. 5b), 2p_3/2_ at 780.0 eV and 2p_1/2_ at 795.0 eV, with a spin-orbital splitting of ~15.0 eV^27^. Two weak shake-up satellite peaks occur at 789.5 and 804.5 eV, which is a fingerprint indication of Co^3+^ cations^28^. By XPS curve fitting, the coexistence of Co^4+^ is confirmed with the existence of two weak peaks at 782.1 and 796.6 eV. The Mn 2p spectrum as shown in Fig. 5c has two major peaks, 2p_3/2_ at 642.8 eV and 2p_1/2_ at 654.5 eV with a separation about 11.7 eV corresponding to Mn^4+^ cation. At 637.5 eV, there is a shoulder related to the Auger peak. Except for the dominant Mn^4+^, a small percentage of lower oxidation state Mn^3+^ also exists (641.8 and 653.2 eV). Figure 5d shows that the Ni 2p spectrum has two dominant peaks located at 854.6 eV (2p_3/2_) and 872.3 eV (2p_1/2_), which represent the Ni^2+^ cations^29^. The presence of two less dominant shake-up peaks further confirms the existence of Ni^2+^ (861 and 879.2 eV). However, weaker peaks at 856.5, 863.9, 874 and 882.4 eV, reveal the existence of the higher oxidation state of Ni^3+^. As reported by Shaju *et al*.^30^, the minor contribution of Ni^3+^ and Mn^3+^ is due to the electron transfer between Ni^2+^ and Mn^4+^, which will cause the valency-degeneracy: Mn^4+^ +Ni^2+^ ↔ Ni^3+^+Mn^3+^.

**Figure 6:**
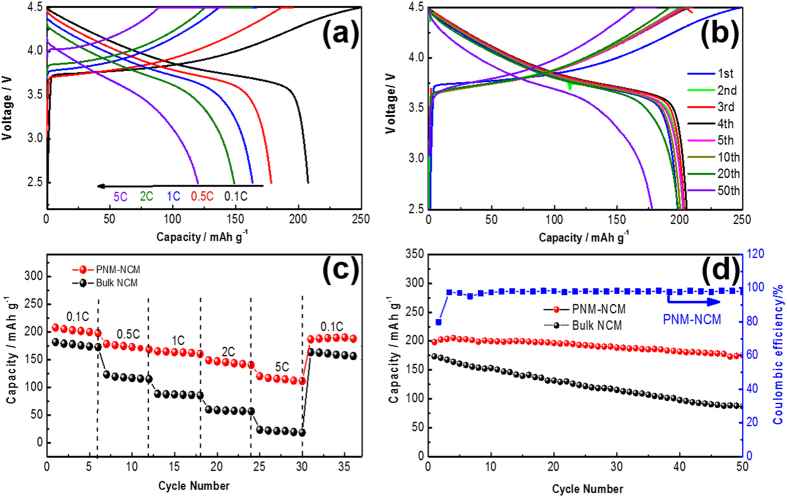
Electrochemical characterization of the PNM-NCM electrode as the cathode of lithium ion batteries: (**a**) the charge and discharge curves at different C-rates; (**b**) the charge and discharge curves for different cycle at 0.1 C; (**c**) cycling behavior at various current densities; (**d**) cycling behaviors at 0.1 C.

To evaluate the rate performance of NCM microspheres, the PNM-NCM sample was tested at various current densities (0.1–5 C, 1 C = 200 mA g^−1^). The cells were firstly charged galvanostaticly and followed by a constant voltage charge process. For each cell, the average active material loading density is about 2.0 ± 0.4 mg cm^−2^. From Fig. 6a, the initial capacity is 207.7 mAh g^−1^ at 0.1 C, and with increasing the charging current density, the capacity slightly drops to 178.4 (0.5 C), 163.6 (1 C), 148.9 (2 C) and 120.0 (5 C), respectively. As for the cycling performance (tested at 0.1 C, as shown in Fig. 6b), the capacity of initial 5 cycles is 198.2, 202.5, 203.0, 205.5 and 203.6 mAh g^−1^, respectively. After 10, 20 and 50 cycles, the capacity slightly drops to 200.4, 198.7 and 179.0 mAh g^−1^, respectively. The capacity retention after 50 cycles remains as high as 90.3%. Figure 6c,d show a comparison of the rate capability and cycling performance between PNM-NCM and Bulk-NCM. Compared with PNM-NCM, the capacity of the Bulk-NCM electrode are only 181.8 (0.1 C), 123.1 (0.5 C), 88.3 (1 C), 60.5 (2 C) and 23.6 mAh g^−1^ (5 C), respectively. In addition, the capacity for Bulk-NCM returns only back to 163.4 mAh g^−1^ at 0.1 C with a retention of 90% (compared with 94% capacity retention of PNM-NCM, see Fig. S6†). In terms of cycling performance, the capacity recover ratio after 50 cycles of the PNM-NCM microspheres and Bulk-NCM samples are 90.3% and 50.1% respectively (Fig. 6d). The obvious superiority of PNM-NCM over Bulk-NCM is attributed to the unique highly hierarchical porous nano-/micro structure with smaller grain size, minimized cation mixing of Li^+^/ Ni^2+^ and better crystalline ordering in hexagonal a-b plane.

**Figure 7:**
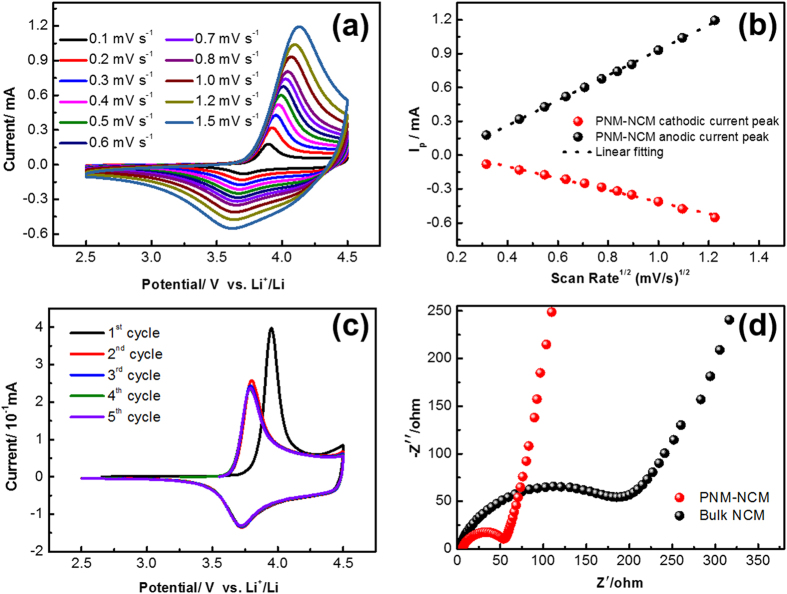
(**a**) Cyclic voltammetric profiles at various scan rates of 0.1–1.5 mV s^−1^; (**b**) Linear response of the peak current density as a function of the square root of scan rate of PNM-NCM; (**c**) The first five consecutive cyclic voltammograms curves at a scan rate of 0.1 mV s^−1^ in the voltage range 2.5–4.5 V versus Li^+^/Li, (**d**) Nyquist plots over the frequency range from 0.1 kHz to 100 kHz of PNM-NCM (red curve) and Bulk-NCM (black curve) electrodes.

Cyclic voltammetry analyses at various scan rates were carried out to investigate the lithium ion apparent diffusion coefficient, which is a key factor for electrochemical performance^31^. For both PNM-NCM and Bulk-NCM, the anodic peak shifts to higher potential while the cathodic peak shifts to lower potential with increasing scan rate from Figs 7a and S7a†, indicating the diffusion controlled behavior. The peak intensity I_p_ is found to increase with the increasing scan rate. A linear relationship was observed between peak current intensity and the square root of scan rate as shown in Figs 7b and S7b†. According to the Randles-Sevcik equation:


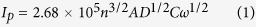


where I_p_ is the peak current intensity (A), n is the number of electrons per reaction species, A is the electrode area (1.13 cm^2^), D is the apparent diffusion coefficient of lithium ions (cm^2^ s^−1^), C is the molar concentration of lithium ions in NCM (6.55*10^−2^ mol cm^−3^) and the ω is the scan rate (mV s^−1^). The lithium ion apparent diffusion coefficients, which are 3.126*10^−9^ and 6.921*10^−10^ cm^2^ s^−1^ for delithiation and lithiation processes of PNM-NCM (Fig. 7b), while 1.556*10^−9^ and 3.548*10^−10^ cm^2^ s^−1^ for Bulk-NCM (Fig. S7b†), can be obtained from the slope of 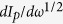. The apparent diffusion coefficient is comparable to previous reports (see Supplementary Table 3†). Besides the minimized cation mixing, the improved Li mobility of PNM-NCM sample also contributes a lot to the superior electrochemical performance compared with Bulk-NCM.

Figures 7c and S8† show the cyclic voltammograms of the electrode made of PNM-NCM and Bulk-NCM over the voltage range from 2.5 to 4.5 V with a scanning rate of 0.1 mV s^−1^. In the first cycle, the anodic and cathodic peaks occur at 3.950 and 3.732 V with a separation around 0.218 V. However, after the 1^st^ cycle, the redox peak separation becomes much smaller, i.e. 0.071 V for the 2^nd^ cycle and 0.062 V for the 3^rd^–5^th^ cycles respectively (see Supplementary Table 4†), which are very close to the theoretical value 0.059 V (i.e. 2.303RT/F) for a reversible redox process at room temperature. The large redox peak separation (0.218 V) in the first charge/discharge cycle can be attributed to the polarization due to anti-site Li^+^/Ni^2+^ mixing. This value is reduced to ~0.06 V in the 2^nd^ cycle onwards, which may be indicative of mitigation of anti-site defects. An *in-situ* neutron diffraction study has recently revealed that the Li ions in the anti-sites of metal oxide layers are preferentially extracted^32^. Likewise the small redox peak separation and high reversibility observed in Fig. 6c in the 2^nd^–5^th^ cycles appear to prove the maintaining of minimized Li^+^/Ni^2+^ mixing in PNM-NCM after the anti-site Li ions are removed from the metal oxygen layers in the first charge/discharge cycle. The ~100% reversibility obtained in our PNM-NCM is exceptional (to our best knowledge never reported), which is indicative of minimized Li^+^/Ni^2+^ mixing. In contrast, for Bulk-NCM sample measured under identical measurement conditions, a large redox peak separation is observed in the initial five cycles (0.321, 0.377, 0.310, 0.276 and 0.263 V, respectively) as shown in Fig. S8† and supplementary Table 4†. Similarly, a value of 0.2 V (or much bigger value) for the redox peak separation is usually reported for the NCM electrode in the first cycle and remaining cycles (see Supplementary Table 5†)^25^. The much smaller redox peak separation of PNM-NCM (~0.06 V vs. 0.26 V of Bulk-NCM) appears to benefit from the hierarchical porous nano-/micro structure, the optimized crystallization condition (high hexagonal ordering in a-b plane) and minimized cation mixing of Li^+^/Ni^2+^.

The Nyquist plots of the PNM-NCM microspheres and Bulk-NCM electrodes tested by electrochemical impedance spectroscopy (EIS) over the frequency range from 0.1 Hz to 100 kHz are shown in Fig. 7d. Both samples show one semicircular arc at high-medium frequency region and one Warburg tail at low frequency region^33^. The intercept of the semicircle at the high frequency is related to the equivalent internal resistance (R_SEI_), including electrolyte and solid/electrolyte interface resistances. Both PNM-NCM and Bulk-NCM samples reveal a very low internal resistance. However, the intercept at medium frequency region that corresponds to the charge transfer resistance (R_CT_) at the interface of electrolyte and electrode is less than 60 Ω for PNM-NCM, which is much less than that (>200 Ω) of Bulk-NCM. In addition, the slope of the Warburg tail for PNM-NCM is much larger than that of Bulk-NCM, indicating higher lithium ion mobility. All these EIS results indicate that the PNM-NCM sample has much higher conductivity and lower polarization, which are contributed to the high hexagonal ordering and minimized cation mixing, resulting in its good electrochemical performance.

## Conclusion

In conclusion, hierarchical porous nano-/micro NCM (PNM-NCM) was synthesized using porous fluffy MnO_2_ as the sacrificial template. The PNM-NCM, which are composed of primary particles with sizes of 56.6 nm, possesses extremely uniform secondary particles with sizes of 3 μm. The desired primary particle sizes have been proven mostly suitable for NCM to provide high atomic ordering for Li^+^ diffusion. Cyclic voltammetry shows a minimum redox peak separation for PNM-NCM (~0.06 V vs. 0.26 V for Bulk-NCM), indicating a minimized cation mixing in agreement with our XRD studies and hence an improved Li^+^ mobility. The excellent rate capability and cycling performance observed for PNM-NCM are far superior over those of Bulk-NCM: 207.7 (181.8) at 0.1 C, 163.6 (88.3) at 1 C, 120.0 mAh g^−1^ (23.6) at 5 C and 90.3% (50.1%) capacity retention after 50 cycles. The enhancement is attributed to the hierarchical porous nano-/micro structure of PNM-NCM, which can largely shorten the diffusion path of lithium ions, enhance the Li mobility, alleviate volume expansion/compression during the discharge/charge processes and stabilize the whole structure of the electrode. In addition, PNM-NCM reveals the minimized anti-site mixing of Li^+^/Ni^2+^ and high ordering hexagonal arrangement in a-b plane, resulting in the exceptionally high reversibility, low polarization and thus excellent electrochemical performance. Our detailed study fundamentally establishes a relationship between the chemistry of hierarchical nano-/micro structures and their properties. We expect that the strategy developed in this study may open a new way to scale up traditional materials for LIBs.

## Experimental Section

### Preparation of PNM-NCM

Firstly, porous fluffy manganese dioxide (MnO_2_) which serves as a sacrificial template for PNM-NCM, was synthesized by a co-precipitation method using silver nitrate as a catalyst. In brief, 2.704 g of manganese sulfate monohydrate (MnSO_4_·H_2_O) and 3.650 g of ammonium persulfate ((NH_4_)_2_S_2_O_8_) were dissolved in 200 ml of DI water under continuous stirring until it became transparent solution. After that, 8 ml of concentrated sulfuric acid was added dropwise into the above transparent solution, followed by the addition of silver nitrate (0.06 M AgNO_3_) under rigorous stirring. After aging for 24 h in a fume cupboard, the obtained mixture was centrifuged, and washed several times with DI water and ethanol respectively, until the pH of solution became neutral. The mixture was then dried at 80 °C in a vacuum oven overnight. The as-prepared MnO_2_ was dispersed in 40 ml of ethanol, together with stoichiometric Ni(NO_3_)_2_·6H_2_O, Co(NO_3_)_2_·6H_2_O and LiOH·H_2_O (6% excess), under continuous stirring overnight until the ethanol was completely evaporated. The powder was then collected and calcined in a tube furnace at 900 °C for 12 h in air.

### Preparation of Bulk-NCM

For comparison, bulk LiNi_1/3_Co_1/3_Mn_1/3_O_2_ (Bulk-NCM) was prepared by co-precipitation method as reported in literature^24^. In brief, (Ni_1/3_Co_1/3_Mn_1/3_)(OH)_2_ was firstly synthesized at room temperature through a co-precipitation method, followed by a solid state reaction with LiOH.H_2_O at high temperature.

### Material Characterizations

XRD patterns of both PNM-NCM and Bulk-NCM samples were identified using X-ray diffraction (XRD, Bruker D8 with Cu Kα radiation, λ = 0.15406 nm). The morphology of the samples was observed by field emission scanning electron microscopy (FESEM, JSM-7600F, JEOL). The elemental compositions of the PNM-NCM sample were characterized using energy dispersive spectroscopy (EDS) (Oxford INCA, England). The nanostructure of the samples was investigated using a high resolution transmission electron microscope (HRTEM, JEOL JEM-2010F) operating at 200 kV. The thermal Brunauer-Emmett-Teller (BET) surface area and porosity were determined by nitrogen-sorption using a Micromeritics ASAP 2020 analyzer. The X-ray photoelectron spectroscopy (XPS) measurements were performed with a VG ESCALAB 220i-XL system using a monochromatic Al Kα source (1486.6 eV) to investigate the oxidation states of the Ni, Co and Mn. Gas sorption analyses were conducted using Quantachrome Instruments Autosorb-iQ (Boynton Beach, Florida USA) with extra-high pure gases.

### Electrochemical Measurements

Electrochemical measurements were carried out using CR2032 coin cells. The working electrodes were prepared with a loading density of 2.0 ± 0.4 mg cm^−2^. The electrodes were prepared by casting slurries comprising of 80 wt% NCM sample, 10 wt% carbon black and 10 wt% poly-(vinylidene fluoride) (PVDF) binder dissolved in N-methyl-2-pyrrolidone (NMP) on aluminum foil, followed by drying in a vacuum oven at 100 °C overnight. The cells were assembled in an argon-filled glove box (with O_2_ <0.1 ppm and H_2_O <0.1 ppm) using metallic lithium as the reference electrode, Celgard 2400 films as the separator and 1 M LiPF_6_ dissolved in ethyl carbonate–dimethyl carbonate (EC–DMC) (1:1 v/v) as the electrolyte. The charge–discharge testing was performed galvanostatically at different C-rates between 2.5 and 4.5 V on a LAND CT-2001A battery tester. Cyclic voltammetry (CV) curve (at a scan rate of 0.1 mV s^−1^) as well as CV curves at various scan rates (0.1–1.5 mV s^−1^) were conducted on CHI750d electrochemical workstation between the potential range of 2.5–4.5 V (vs. Li^+^/Li). Electrochemical impedance spectroscopic (EIS) measurements were carried out using two-electrode coin cells at room temperature with CHI760d electrochemical workstation over the frequency range between 0.1 Hz and 100 kHz.

## Additional Information

**How to cite this article**: Chen, Z. *et al*. Hierarchical Porous LiNi_1/3_Co_1/3_Mn__1/3__O_2_ Nano-/Micro Spherical Cathode Material: Minimized Cation Mixing and Improved Li^+^ Mobility for Enhanced Electrochemical Performance. *Sci. Rep.* **6**, 25771; doi: 10.1038/srep25771 (2016).

## Supplementary Material

Supplementary Information
