# Supplementary material for: Hierarchical Porous LiNi1/3Co1/3Mn1/3O2 Nano-/Micro Spherical Cathode Material: Minimized Cation Mixing and Improved Li+ Mobility for Enhanced Electrochemical Performance
Source: Sci Rep. 2016 May 17;6:25771. doi: 10.1038/srep25771 (PMC4868989; doi:10.1038/srep25771)
Supplement: Supplementary Information [file srep25771-s1.pdf]

Supporting Information

Hierarchical Porous $LiNi_{1/3}Co_{1/3}Mn_{1/3}O_2$ Nano-/Micro Spherical Cathode Material: Minimized Cation Mixing and Improved Li^+ Mobility for Enhanced Electrochemical Performance

Zhen Chen[1,2], Jin Wang[1,2], Dongliang Chao[2], Tom Baikie[3], Linyi Bai[4], Shi Chen[2], Yanli Zhao[4, 5], Tze Chien Sum[2], Jianyi Lin[3, *], Zexiang Shen[2,3, *]

1Energy Research Institute (ERI@N), Interdisciplinary Graduate School, Nanyang Technological University, Research Techno Plaza, 50 Nanyang Drive, 637553, Singapore

2Division of Physics and Applied Physics, School of Physical and Mathematical Sciences, Nanyang Technological University, 21 Nanyang Link, 637371, Singapore

3Energy Research Institute (ERI@N), Nanyang Technological University, 50 Nanyang Drive, 637553, Singapore

4Division of Chemistry and Biological Chemistry, School of Physical and Mathematical Sciences, Nanyang Technological University, 21 Nanyang Link, 637371,Singapore

5School of Materials Science and Engineering, Nanyang Technological University, 639798, Singapore

*** Email: zexiang@ntu.edu.sg, Lijy@ntu.edu.sg**

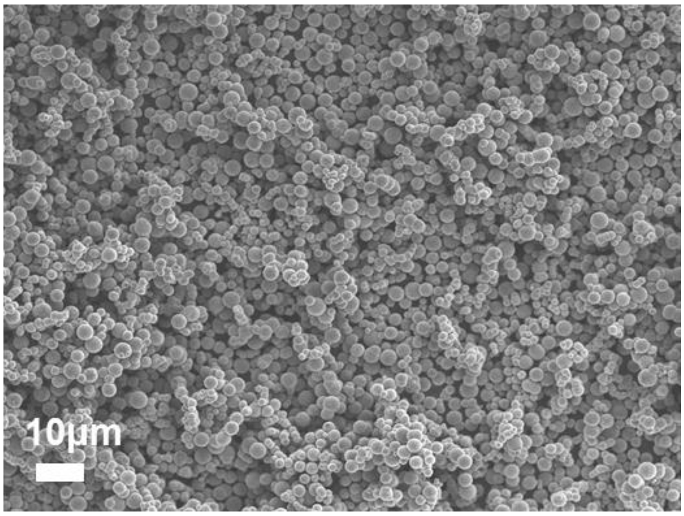

Figure S1. FESEM image of porous fluffy MnO_2.

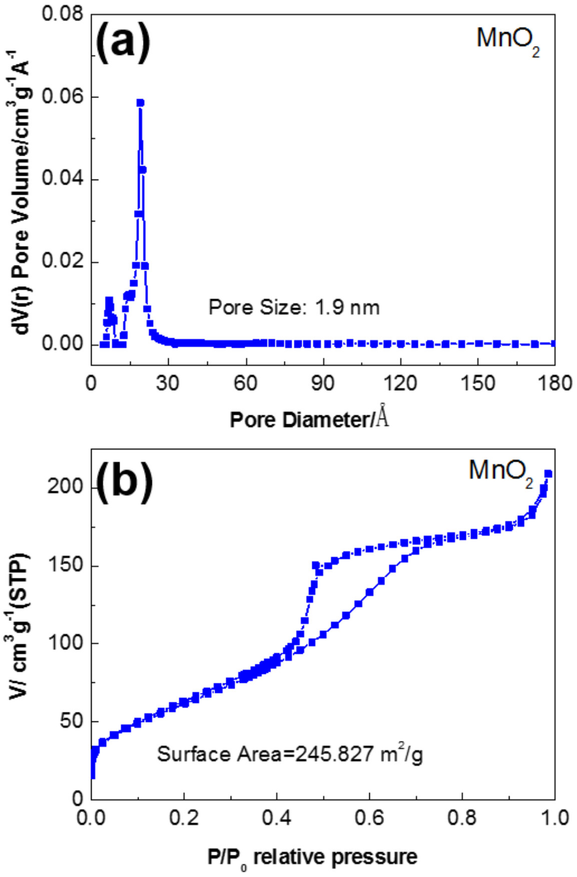

Figure S2. (a) The pore size distribution, and (b) N_2 adsorption/desorption isotherms of fluffy MnO_2.

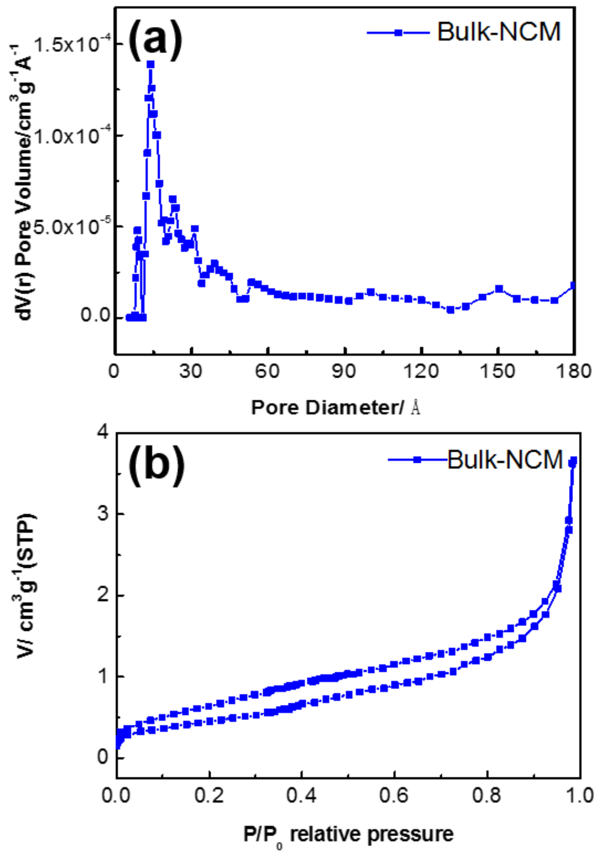

Figure S3. (a) The pore size distribution, and (b) N_2 adsorption/desorption isotherms of Bulk-NCM.

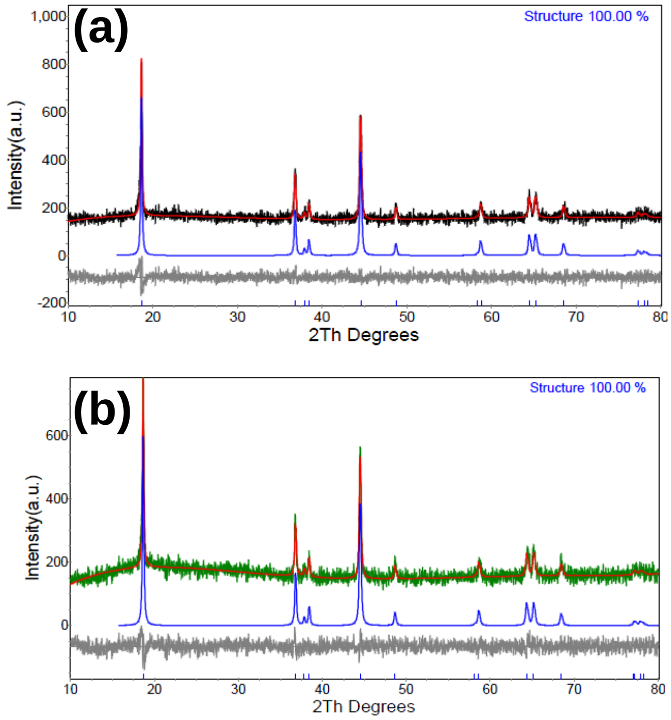

Figure S4. Rietveld refinement of the XRD data for (a) PNM-NCM; (b) Bulk NCM.

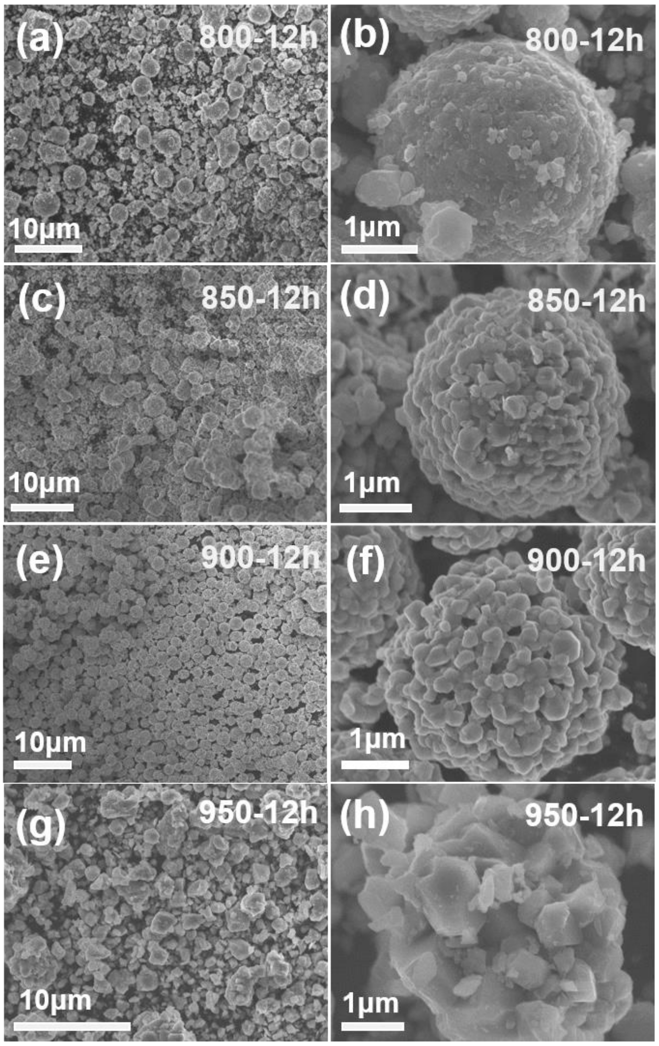

Figure S5. FESEM images of NCM microspheres calcined at different temperature: (a) and (b) 800 ^{o}C; (c) and (d) 850 ^{o}C; (e) and (f) 900 ^{o}C; (g) and (h) 950 ^{o}C.

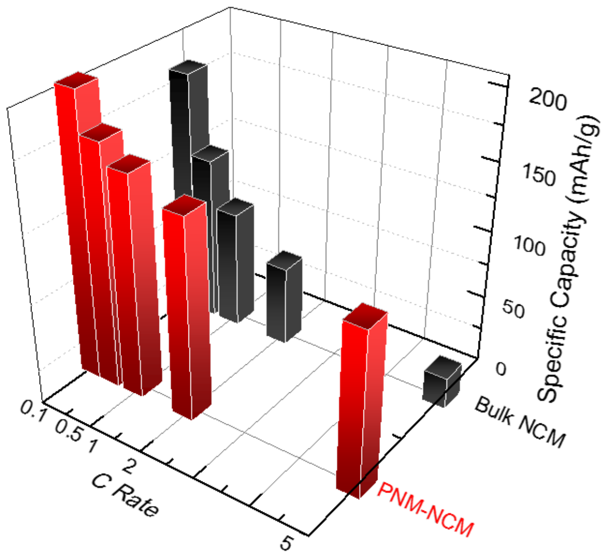

Figure S6. The comparisons of rate capability of electrodes made by PNM-NCM (red columns) and Bulk-NCM (black columns).

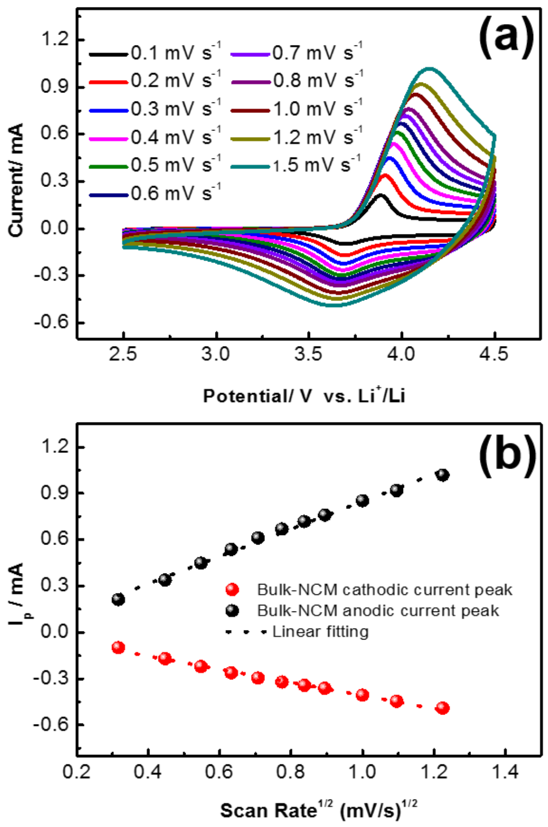

Figure S7. (a) Cyclic voltammetric profiles at various scan rates of 0.1-1.5 mV s^{-1}, (b) Linear response of the peak current density as a function of the square root of scan rate of Bulk-NCM.

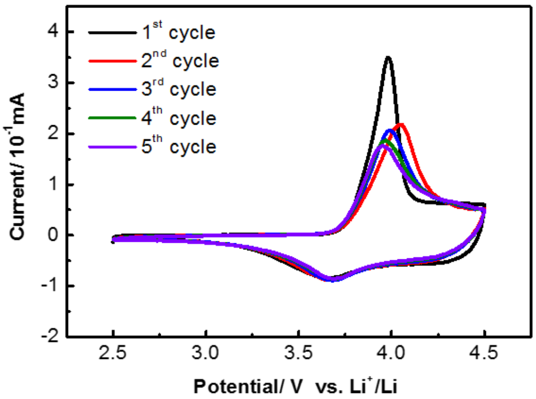

Figure S8. The first five consecutive cyclic voltammograms curves at a scan rate of 0.1 mV s^{-1} over the voltage range 2.5-4.5 V versus Li^{+}/Li of Bulk-NCM.

Table 1. Summary of PNM-NCM Refinement

Cell Parameters

Space Group R3m

a=2.860(2)Å; c=14.216(8) Å; V=100.50 Å^3; Z=3

I(003)/I(103)=1.399; R=0.875; crystal size=56.6nm，

R-factor=0.52

Atomic Positions

site	x	y	z	atom	fract
3a	0.00000	0.00000	0.00000	Li^{1+}	0.981
3a	0.00000	0.00000	0.00000	Ni^{2+}	0.019
3b	0.00000	0.00000	0.50000	Li^{1+}	0.019
3b	0.00000	0.00000	0.50000	Ni^{2+}	0.314
3b	0.00000	0.00000	0.50000	Co^{3+}	0.333
3b	0.00000	0.00000	0.50000	Mn^{4+}	0.333
6c	0.00000	0.00000	0.26030	O^{2-}	1

Table 1. XRD pattern refinement parameters of PNM-NCM microspheres.

Table 2. Summary of bulk NCM Refinement

Cell Parameters

Space Group R3m

a=2.865(2)Å; c=14.281(8) Å; V=101.50 Å^3; Z=3

I(003)/I(103)=1.484; R=1.583; crystal size=97.0nm，

R-factor=0.58

Atomic Positions

site	x	y	z	atom	fract
3a	0.00000	0.00000	0.00000	Li^{1+}	0.957
3a	0.00000	0.00000	0.00000	Ni^{2+}	0.043
3b	0.00000	0.00000	0.50000	Li^{1+}	0.057
3b	0.00000	0.00000	0.50000	Ni^{2+}	0.276
3b	0.00000	0.00000	0.50000	Co^{3+}	0.333
3b	0.00000	0.00000	0.50000	Mn^{4+}	0.333
6c	0.00000	0.00000	0.26030	O^{2-}	1

Table 2. XRD pattern refinement parameters of Bulk NCM.

Reference	Delithiation/ cm^2 s^{-1}	Lithiation/ cm^2 s^{-1}
S1[1]	$1.39*10^{-10}$	$1.51*10^{-10}$
S2[2]	$4.4*10^{-10}$	$2.6*10^{-10}$
S3[3]	$6.6*10^{-10}$	$3.9*10^{-10}$
S4[4]	$2.04*10^{-8}$	$6.12*10^{-9}$
S5[5]	$7.8*10^{-10}$	$4.3*10^{-10}$
This work	$3.126*10^{-9}$	$6.921*10^{-10}$

Table 3. Comparison of apparent diffusion coefficient of lithium ions among the references.

PNM-NCM	1st cycle	2nd cycle	3rd cycle	4th cycle	5th cycle
Oxidation Peak	3.950	3.798	3.792	3.788	3.792
Reduction Peak	3.732	3.727	3.730	3.724	3.720
**Bulk NCM**	**1st cycle**	**2nd cycle**	**3rd cycle**	**4th cycle**	**5th cycle**
Oxidation Peak	3.980	4.045	3.992	3.966	3.953
Reduction Peak	3.668	3.668	3.682	3.690	3.690

Table 4. Peak positions of the initial five consecutive cyclic voltammetric curves of PNM-NCM and bulk-NCM microspheres at a scan rate of 0.1 mV s^{-1} in the voltage range 2.5-4.5 V versus Li^+/Li.

Reference	Year	Journal	Redox Peak Separation Value
S1[6]	Sept, 2010	Scripta Materialia	~0.2V
S2[3]	May, 2011	J. Mater. Chem.	~0.3V
S3[4]	Jun, 2013	Nano Energy	~0.2V
S4[7]	Jul, 2013	J. Mater. Chem.A	~0.23V
S5[8]	Feb, 2014	Journal of Power Sources	~0.1V
S6[9]	Mar, 2014	ACS Appl. Mater. Interfaces	~0.18V
S7[10]	Aug, 2014	Journal of Power Sources	~0.23V
S8[11]	Jul, 2015	J. Mater. Chem.A	~0.27V
S9[5]	Jul, 2015	Nano Energy	~0.2V
S10[12]	Sept, 2015	ACS Appl. Mater. Interfaces	~0.3V

Table 5. Comparison of redox peak separation values among the references.

References in ESI:

1 Zheng, J. *et al.* Electrochemical Performance of the $LiNi_{1/3}Mn_{1/3}Co_{1/3}O_2$ in Aqueous Electrolyte. *J. Electrochem. Soc.* **157**, A702, doi:10.1149/1.3374663 (2010).

2 Zhang, X. *et al.* Synthesis and characterization of $LiNi_{1/3}Mn_{1/3}Co_{1/3}O_2$ by wet-chemical method. *Electrochim. Acta* **55**, 6440-6449, doi:http://dx.doi.org/10.1016/j.electacta.2010.06.040 (2010).

3 Huang, Z.-D. *et al.* Microscopically porous, interconnected single crystal $LiNi_{1/3}Mn_{1/3}Co_{1/3}O_2$ cathode material for Lithium ion batteries. *J. Mater. Chem.* **21**, 10777-10784, doi:10.1039/C1JM00059D (2011).

4 Li, J., Xiong, S., Liu, Y., Ju, Z. & Qian, Y. Uniform $LiNi_{1/3}Mn_{1/3}Co_{1/3}O_2$ hollow microspheres: Designed synthesis, topotactical structural transformation and their enhanced electrochemical performance. *Nano Energy* **2**, 1249-1260, doi:http://dx.doi.org/10.1016/j.nanoen.2013.06.003 (2013).

5 Peng, L., Zhu, Y., Khakoo, U., Chen, D. & Yu, G. Self-assembled $LiNi_{1/3}Mn_{1/3}Co_{1/3}O_2$ nanosheet cathodes with tunable rate capability. *Nano Energy* **17**, 36-42, doi:10.1016/j.nanoen.2015.07.031 (2015).

6 Huang, Z.-D. *et al.* $LiNi_{1/3}Mn_{1/3}Co_{1/3}O_2$ with a novel one-dimensional porous structure: A high-power cathode material for rechargeable Li-ion batteries. *Scr. Mater.* **64**, 122-125, doi:http://dx.doi.org/10.1016/j.scriptamat.2010.09.018 (2011).

7 Li, J., Cao, C., Xu, X., Zhu, Y. & Yao, R. $LiNi_{1/3}Mn_{1/3}Co_{1/3}O_2$ hollow nano-micro hierarchical microspheres with enhanced performances as cathodes for lithium-ion batteries. *J. Mater. Chem. A* **1**, 11848-11852, doi:10.1039/C3TA12375H (2013).

8 Ryu, W.-H., Lim, S.-J., Kim, W.-K. & Kwon, H. 3-D dumbbell-like $LiNi_{1/3}Mn_{1/3}Co_{1/3}O_2$ cathode materials assembled with nano-building blocks for lithium-ion batteries. *J. Power Sources* **257**, 186-191, doi:http://dx.doi.org/10.1016/j.jpowsour.2014.02.003 (2014).

9 Li, J., Yao, R. & Cao, C. $LiNi_{1/3}Mn_{1/3}Co_{1/3}O_2$ Nanoplates with {010} Active Planes Exposing Prepared in Polyol Medium as a High-Performance Cathode for Li-Ion Battery. *ACS Appl. Mater. Interfaces* **6**, 5075-5082, doi:10.1021/am500215b (2014).

10 Yang, Z. *et al.* Stepwise co-precipitation to synthesize $LiNi_{1/3}Mn_{1/3}Co_{1/3}O_2$ one-dimensional hierarchical structure for lithium ion batteries. *J. Power Sources* **272**, 144-151, doi:http://dx.doi.org/10.1016/j.jpowsour.2014.08.052 (2014).

11 Wu, Y., Cao, C., Zhu, Y., Li, J. & Wang, L. Cube-shaped hierarchical $LiNi_{1/3}Mn_{1/3}Co_{1/3}O_2$ with enhanced growth of nanocrystal planes as high-performance cathode materials for lithium-ion batteries. *J. Mater. Chem. A* **3**, 15523-15528, doi:10.1039/c5ta03225c (2015).

12 Li, L. *et al.* Structural and Electrochemical Study of Hierarchical $LiNi_{1/3}Mn_{1/3}Co_{1/3}O_2$ Cathode Material for Lithium-Ion Batteries. *ACS Appl. Mater. Interfaces* **7**, 21939-21947, doi:10.1021/acsami.5b06584 (2015).
